# Case report: Isolated brainstem-cerebellar symptoms in a patient with anti-NMDA receptor encephalitis

**DOI:** 10.3389/fimmu.2024.1388667

**Published:** 2024-05-10

**Authors:** Yongfeng Xu, Qingqing Tao, Yi Dong, Yinxi Zhang

**Affiliations:** Department of Neurology, Second Affiliated Hospital, School of Medicine, Zhejiang University, Hangzhou, China

**Keywords:** Isolated brainstem-cerebellar symptoms, anti-NMDAR encephalitis, encephalitis, ataxia, brainstem symptoms

## Abstract

Cerebellar ataxia is an uncommon and atypical manifestation of anti-*N*-methyl-D-aspartate receptor (NMDAR) encephalitis, often accompanied by seizures, psychiatric symptoms, and cognitive deficits. Previous cases of isolated brainstem-cerebellar symptoms in patients with anti-NMDAR encephalitis have not been documented. This report presents a case of anti-NMDAR encephalitis in which the patient exhibited cerebellar ataxia, nystagmus, diplopia, positive bilateral pathological signs, and hemiparesthesia with no other accompanying symptoms or signs. The presence of positive CSF anti-NMDAR antibodies further supports the diagnosis. Other autoantibodies were excluded through the use of cell-based assays. Immunotherapy was subsequently administered, leading to a gradual recovery of the patient.

## Introduction

Anti-*N*-methyl-D-aspartate receptor (NMDAR) encephalitis is a form of autoimmune encephalitis that typically responds well to immunotherapy, as reported by Titulaer et al. Ovarian teratomas are identified as the underlying pathogenesis in approximately one-third of patients with this condition (1). Common clinical manifestations of the disease include neuropsychiatric symptoms, language dysfunction, altered consciousness, seizures, dyskinesias, dysautonomia, and central hypoventilation (1). In pediatric cases, cerebellar ataxia, speech dysfunction, and hemiparesis are more prevalent, often presenting alongside other symptoms (1). In the adult population, cerebellar ataxia presenting as the initial symptom is uncommon and typically occurs in conjunction with psychiatric symptoms and cognitive impairment (2). The occurrence of isolated brainstem-cerebellar symptoms in patients with anti-NMDAR encephalitis has not been previously documented. This case study presents a unique instance of a woman with anti-NMDAR encephalitis displaying isolated brainstem-cerebellar symptoms.

## Case presentation

A healthy 36-year-old woman experienced right-sided numbness, double vision, and difficulty walking. One month prior to admission, the patient experienced initial symptoms of numbness in the distal end of the lower right extremity, which progressed upwards over time. Approximately 5 days later, the patient reported numbness in the right upper limb, starting from the palm of the hand and advancing proximally. This was followed by the development of facial numbness. After approximately 7 days, the patient began to experience difficulty walking, resembling a drunken gait, which worsened to the point of being unable to walk unassisted. Additionally, the patient gradually developed instability in writing with the right hand, as well as bilateral holding instability, with the right hand particularly affected. The patient presented with normal left limb and facial sensation. Approximately 10 days prior to admission, the patient experienced double vision that worsened gradually, with the absence of double vision when viewing with one eye. Throughout this progression, there were no reported symptoms of headache, fever, mental disorders, memory loss, speech or swallowing difficulties, or incontinence.

Neurological exam revealed sensory abnormalities on the right limbs and face, diplopia, downbeat nystagmus, ataxia, and difficulty walking. Positive Babinski’s sign was present bilaterally. The Mini-Mental State Examination Scale (MMSE) and the Montreal Cognitive Assessment (MoCA) score were both 29/30.

Contrast-enhanced brain magnetic resonance imaging (MRI), electroencephalogram (EEG), and chest computed tomography (CT) revealed no abnormalities. Lumbar puncture indicated normal pressure. Cerebrospinal fluid (CSF) analysis and extensive laboratory investigations showed pleocytosis (**Table 1**). Anti-NMDAR antibodies were positive in CSF (cell-based assay, EUROIMMUN product), but negative in serum. A pelvic MRI revealed bilateral benign cystic lesions in the adnexa. The patient received treatment with intravenous immunoglobulin at a dose of 0.4 g/kg per day for 5 days, in combination with intravenous methylprednisolone at a daily dose of 500 mg for 5 days, followed by an oral therapeutic regimen.

**Table 1:** Overview of investigations.

CSF analysis	Time	
White blood cells	Admission	*100 [ref: 0–8]/µL*
Protein	Admission	*104.7 [ref: 15–45] mg/dL*
Glucose	Admission	2.77 [ref: 2.5–4.4] mmol/L
Gram stain and culture	Admission	Negative
CSF NGS	Admission	Negative
IgG index	Admission	*1.39 [ref: ≤0.7]*
Oligoclonal bands	Admission	*Present*
Cytology testing	Admission	Negative
Laboratory tests	Admission	
Complete blood cell count, blood culture, levels of calcium, phosphorus, magnesium, liver function, thyroid-stimulating hormone, antinuclear antibody, ANCA, anti-thyroid peroxidase antibodies, and anti-thyroglobulin	Admission	Within normal limits
Antibodies in serum and CSF		
Anti-NMDAR in CSF (cell-based assay)	Admission	*1:10*
Anti-NMDAR in serum	Admission	Negative
Anti-AMPAR. anti-LGI1, anti-CASPR2, anti-GABAbR, anti-DPPX, anti-IgLON5, anti-mGluR5, anti-MOG, anti-AQP4, anti-MBP, anti-flotillin-1/2, and anti-GFAP	Admission	Negative
Serum paraneoplastic autoantibodies including anti-Hu, anti-Yo, anti-Ri, anti-Ma2, anti-CV2, anti-amphiphysin, anti-ANNA-3, anti-Tr, anti-PCA2, and anti-GAD65	Admission	Negative
Serum antibodies including anti-VGCC, anti-GQ1b, anti-ATP13A, anti-ARHGAP26, anti-NCDN, anti-Homer3, anti-KLHL11, anti-PCA2 (MAP1B), anti-DNER, anti-ITPR1, and anti-CARPVIII	Admission	Negative
CSF analysis		
White blood cells	Follow-up	*20 [ref: 0–8]/µL*
Protein	Follow-up	34 [ref: 15–45] mg/dL
Glucose	Follow-up	3.76 [ref: 2.5–4.4] mmol/L
Oligoclonal bands	Follow-up	*Present*
Antibodies in serum and CSF	Follow-up	
Anti-NMDAR in CSF (cell-based assay)	Follow-up	*1:3.2*
Anti-NMDAR in serum	Follow-up	Negative

Follow-up was conducted 1 month after the initial assessment.

NGS, Next-Generation Sequencing; ANCA, Anti-Neutrophil Cytoplasmic Antibody; NMDAR, N-Methyl-D-Aspartate Receptor; AMPAR, α-Amino-3-hydroxy-5-Methyl-4-isoxazole-Propionic acid 1 Receptor; LGI1, Leucine-rich Glioma Inactivated-1 (LGI1); CASPR2, Contactin-Associated Protein-like Receptor 2 (CASPR2); GABAbR, γ-Aminobutyric Acid-B Receptor; DPPX, Dipeptidyl-Peptidase-like Protein 6; mGluR5, metabotropic glutamate Receptor 5; MOG, Myelin Oligodendrocyte Glycoprotein; AQP4, Aquaporin 4; MBP, Monobutyl Phthalate; GFAP, Glial Fibrillary Acidic Protein; ANNA-3, Anti-Neuronal Nuclear Antibody-type 3; PCA2, Purkinje cell Cytoplasmic Antibody type 2; GAD65, Glutamic Acid Decarboxylase-65; VGCC, Voltage-Gated Calcium Channel; ATP13A, ATPase type 13A; ARHGAP26, RhoA GTPase-Activating Protein 26; NCDN, Neurochondrin; KLHL11, Kelch-like protein 11; MAP1B, Microtubule-Associated Protein 1B (MAP1B); DNER, Delta/Notch-like EGF-related Receptor; ITPR1= Inositol 1;4;5-trisphosphate receptor type 1; CARPVIII, Carbonic Anhydrase-Related Protein VIII.

Abnormal values are shown in italic font.

One month post-treatment, the patient was assessed for functional abilities. The patient demonstrated independent ambulation, albeit with a sense of instability, and was able to ascend stairs normally but descended at a slower pace. Manual dexterity, particularly in holding objects with both hands, showed improvement, with the right hand demonstrating the ability to write. Sensory deficits persisted on the right side of the face and limbs, accompanied by intermittent electric shock-like sensations, albeit with reduced frequency. While diplopia was absent during forward gaze, it persisted when looking to the right. The physical examination revealed the absence of significant nystagmus, normal legibility of his name, and continued presence of bilateral pathological signs. The lumbar puncture was repeated, and CSF white blood cell count decreased (**Table 1**). Additionally, the presence of a positive CSF antibody against NMDAR (cell-based assay, CBA, EUROIMMUN product) was noted, while serum antibody against NMDAR was negative. Oligoclonal bands (OBs) were detected in the CSF sample. The patient was advised to maintain oral corticosteroid therapy with tapering and was prescribed mycophenolate mofetil. Instructions were given for regular monitoring of teratoma. A recent follow-up by phone indicated the patient’s stable condition, although the patient continued to experience numbness in the right limb and unstable walking, with minimal impact on daily activities. Following our recommendation, the patient underwent whole-body positron emission tomography (PET)-CT scan at a local hospital, which revealed no evidence of tumor presence.

## Discussion

This case report details a patient presenting solely with brainstem-cerebellar symptoms in the context of anti-NMDAR encephalitis, following the exclusion of central nervous system infection and carcinomatous meningitis. While the possibility of co-occurring autoimmune brainstem-cerebellar syndromes cannot be definitively ruled out, it is deemed improbable.

MRI abnormalities of the cerebellum have been documented in 6% of individuals with anti-NMDAR encephalitis (3). Additionally, Iizuka et al. reported that two patients with anti-NMDAR encephalitis exhibited progressive cerebellar atrophy on MRI, which was correlated with a poor clinical prognosis (4). Furthermore, [123I] CLINDE-SPECT imaging has shown a significant increase in binding to the translocator protein 18 kDa (TSPO) in the brainstem and cerebellum of patients with anti-NMDAR encephalitis. TSPO, found on activated microglia, may serve as a marker of regional neuroinflammation (5). Only a small percentage of patients, approximately 5%, exhibit cerebellar complaints, the etiology of which remains unclear. Turner et al. documented a case of a 28-year-old man presenting with a rapidly fatal form of progressive encephalomyelitis with rigidity and myoclonus (PERM). Postmortem analysis revealed the presence of serum antibodies targeting both NMDAR and glycine receptors, suggesting a potential pathogenic role for these antibodies. The authors further observed direct interaction between CD8^+^ cytotoxic T cells and granule cell neurons of the hippocampus and Purkinje cells (6).

In the cerebellum, Purkinje cells typically lack expression of NMDA receptors, while granule cells exhibit NMDA receptor expression. The cerebellar input layer is composed of granule cells, which represent the most abundant neuronal subtype in the brain. Axons of granule cells ascend to bifurcate into parallel fibers along the mediolateral axis, ultimately making contact with the dendrites of Purkinje cells (7). Traditional theories of cerebellar motor learning have historically emphasized plasticity at the parallel fiber-to-Purkinje cell synapse, neglecting the role of granule cells. However, recent research has demonstrated that granule cells are heavily involved in movement and sensory processing (8, 9). Therefore, a comprehensive understanding of cerebellar learning must also consider the contributions of granule cells.

Armangue et al. reported the presence of brainstem-cerebellar symptoms, such as ocular movement abnormalities, opsoclonus–myoclonus syndrome, and low cranial nerve involvement, in patients with ovarian teratomas. However, no anti-NMDAR antibodies were detected in these patients. In contrast, patients with anti-NMDAR encephalitis rarely present with cerebellar complaints, which are typically accompanied by other symptoms (10).

Cerebellar manifestations are predominantly observed in pediatric populations and typically manifest later in the course of the disease. Ataxia is notably uncommon as an initial symptom in adults. Poorthuis et al. documented a case of a woman presenting with ataxia as the initial symptom in the context of anti-NMDAR encephalitis, accompanied by the discovery of ovarian teratomas and subsequent development of psychiatric symptoms and mutism (2). Wang et al. documented dizziness and gait ataxia as the presenting symptoms in a man diagnosed with anti-NMDAR encephalitis. Subsequently, the patient exhibited symptoms of drowsiness, cognitive impairment, and abnormal behavior 2 weeks later. Brain MRI revealed hyperintense lesions affecting the cerebellum (11).

There are certain restrictions in this case. Owing to limitations, CSF rodent brain immunohistochemistry (IHC) was not performed in this study. In cases of anti-NMDAR encephalitis and anti-LGI1 encephalitis presenting with characteristic symptoms (such as anti-NMDAR encephalitis symptoms, faciobrachial dystonic seizures, etc.), a single antibody assay (e.g., CBA) may be adequate for confirmation. However, in instances where symptoms are atypical, it is recommended to confirm antibodies using at least two different techniques. In the case of detecting anti-NMDA receptor antibodies in CSF, IHC is recommended due to the presence of non-typical clinical symptoms. Furthermore, our study did not include testing for mGluR1, mGluR2, GluK2, and various other antibodies, thus limiting our ability to definitively exclude the potential for synergistic effects with other antibodies. Nevertheless, through antibody detection and repeated CSF analysis, we were able to eliminate the likelihood of most other autoimmune encephalitis, infections, and tumors. Ultimately, the positive identification of anti-NMDAR antibodies in the CSF supports our conclusion that the clinical manifestations exhibited by this patient are associated with anti-NMDAR antibodies.

In conclusion, isolated brainstem-cerebellar symptoms have not been reported in adult patients with anti-NMDAR encephalitis. This case shows that anti-NMDAR encephalitis should be included in the list of possible diagnosis when patients present with brainstem-cerebellar symptoms.

## Data availability statement

The original contributions presented in the study are included in the article/supplementary material. Further inquiries can be directed to the corresponding authors.

## Ethics statement

The studies involving humans were approved by Ethics Committee of the Second Affiliated Hospital School of Medicine, Zhejiang University. The studies were conducted in accordance with the local legislation and institutional requirements. The participants provided their written informed consent to participate in this study. Written informed consent was obtained from the individual(s) for the publication of any potentially identifiable images or data included in this article.

## Author contributions

YX: Conceptualization, Writing – original draft, Writing – review & editing. QT: Data curation, Methodology, Validation, Writing – review & editing. YD: Conceptualization, Formal Analysis, Writing – review & editing. YZ: Conceptualization, Formal Analysis, Methodology, Supervision, Validation, Writing – review & editing.
